# Management and prevention of soft tissue complications in implant dentistry

**DOI:** 10.1111/prd.12415

**Published:** 2022-02-01

**Authors:** Daniel S. Thoma, Alfonso Gil, Christoph H. F. Hämmerle, Ronald E. Jung

**Affiliations:** ^1^ Clinic of Reconstructive Dentistry Center of Dental Medicine University of Zurich Zurich Switzerland

**Keywords:** implant mucosa augmentation, peri‐implant biology, peri‐implant mucosa, soft tissue complications

## Abstract

The management and prevention of soft tissue complications is of key importance in modern implant dentistry and influences biologic and esthetic outcomes. The assessment of the soft tissue conditions from a quantitative and qualitative perspective should, therefore, be part of the overall treatment plan. Such an assessment dictates a potential indication as well as an ideal time point for additional soft tissue management. A proper risk assessment and management of the soft tissues at the planned implant site are of key importance prior to any implant‐related surgery. Cases with peri‐implant soft tissue complications generally involve: (a) a lack of attached and keratinized mucosa; (b) insufficient volume; (c) development of mucosal dehiscences; or (d) a combination of (a), (b), and (c). In case of soft tissue deficiencies, these should be addressed as early as possible to increase the predictability of the surgical interventions. This article reviews the main causes for peri‐implant soft tissue complications and presents different therapeutic options for the management of various clinical scenarios.

## 1 INTRODUCTION

Dental implants are frequently used to support fixed and removable prostheses in partially and fully edentulous patients. Based on recent systematic reviews including a plethora of clinical studies, high survival rates can be expected at both the implant and the restorative level. 1, 2

For a long time, implant research was focused on the peri‐implant bone, establishing hard tissue quality and quantity as being the principal criteria for defining success.^3^ Therefore, adequate bone volume was a prerequisite prior to implant placement, with various ridge preservation and augmentation procedures performed accordingly.^4^ A facial bone thickness of at least 2 mm was suggested to maintain marginal bone levels around the implant over time.^5^ Crestal bone loss^6^ and primary implant stability^7^ were considered as the critical factors for success, whereas the importance of the peri‐implant soft tissues was frequently neglected.

More recently, emerging evidence suggests that the peri‐implant soft tissues are key to maintaining peri‐implant health.^8^ Current long‐term clinical studies have shown stable and healthy peri‐implant soft tissues after 7 ^9^ and 12 years ^10^, even in the case of missing buccal bone at implant sites.

Various publications have evaluated the importance of soft tissues at dental implant sites from both a biologic^11^, ^12^, ^13^ and esthetic perspective.^14^, ^15^ Accordingly, various indications^16^, ^17^, ^18^ and treatment options^19^, ^20^ have been suggested.

The present narrative review focuses on the management, timing, specific interventions, and the prevention of soft tissue complications in implant dentistry. Besides the evidential background, this article provides a time line and a risk scale for different interventions to prevent and manage soft tissue complications.

## 2 ANATOMY OF THE PERI‐IMPLANT MUCOSA AND SUSCEPTIBILITY TO INFLAMMATION

The anatomy of the peri‐implant mucosa differs from the gingiva around natural teeth. First, the peri‐implant connective tissue fibers run parallel to the implant surface and, in general, do not attach to it, whereas the dento‐gingival fibers show a perpendicular disposition, attaching directly to the root cementum.^21^ Second, the vascular supply at implant sites is diminished because there is no periodontal ligament present and the only source of nourishment is derived from the supra‐periosteal blood vessels.^22^ Third, the junctional epithelium around implants is more permeable and its connective tissue compartment shows fewer fibroblasts and a greater number of collagen fibers.^23^ These anatomical differences render dental implants more susceptible to inflammation and subsequent bone loss from microbial challenge.^24^ The maintenance of an adequate quantity and quality of mucosa surrounding the peri‐implant bone has been demonstrated to be of paramount importance in maintaining peri‐implant health.^25^

Peri‐implant health is characterized by the absence of bleeding on probing and stable marginal bone levels. Peri‐implant mucositis is defined as the presence of bleeding on probing and/or suppuration but without any evidence of bone loss. Peri‐implantitis requires progressive crestal bone level changes, in addition to bleeding on probing and/or suppuration, with or without deepening of peri‐implant pockets.^26^

The prevention of peri‐implant disease has become a major task in daily practice based on epidemiologic data suggesting that 30% of all implants and 47% of all patients will experience peri‐implant mucositis, and that 10% of all implants and 20% of all patients will experience peri‐implantitis.^27^

Indeed, there is increasing evidence demonstrating that the long‐term maintenance of peri‐implant health is a difficult challenge and that trans‐mucosal healing and adequate management of the peri‐implant mucosa may be a decisive factor in avoiding the development of complications. In addition, a recent systematic review recommended soft tissue augmentation procedures to maintain and improve peri‐implant health at dental implants, suggesting that these procedures limit marginal bone loss and reduce the incidence of bleeding on probing over the long term.^28^

## 3 SOFT TISSUE COMPLICATIONS AROUND IMPLANTS

Three types of soft tissue complications may develop around dental implants and represent an everyday clinical challenge, namely, a lack of attached mucosa, volume deficiency, and peri‐implant mucosal recession.

After tooth extraction, a significant reduction in the ridge dimensions occurs.^29^, ^30^, ^31^ This shrinkage of the alveolar ridge is not limited to the bone but may also be accompanied by a loss of attached tissue and/or a soft tissue volume deficiency.^32^

The incidence of a complete absence of an adequate band of attached and keratinized tissue has been reported to range from 46% to 74% of all inserted implants.^33^ By contrast, incidence data for the lack of mucosal volume around implants has not been reported in the literature because of the difficulties in assessing it in a noninvasive manner. Data, however, suggests that it is a common finding among implants placed in the esthetic zone and its occurrence plays a role in the mucosal color match of implants compared with their adjacent dentition. The color of the peri‐implant tissues matches those of the neighboring teeth in only 33% of cases.^34^ This mismatch is more obvious in thinner biotypes, where a discoloration may still be clinically noticeable.^35^ This, to some extent, underlines the necessity to perform soft tissue grafting procedures, predominantly in the esthetic zone.

The occurrence of recession defects on the buccal side of dental implants is influenced by various factors, such as tissue phenotype, facial bone level, implant angulation and axis, interproximal marginal bone level, implant design, and the level of first bone to implant contact.^36^ The incidence of such recession defects varies, depending on the time point when dental implants were placed. For immediate implants, an advanced recession at 10% of implants has been reported,^37^ whereas for delayed implants, > 1 mm of midfacial soft tissue recession can be expected in 60% of implants.^38^

All three types of soft tissue complication are, therefore, a common clinical finding and may hamper peri‐implant health and the esthetic outcome of implant‐borne reconstructions.

### 3.1 Attached mucosa

The influence of a sufficient width of attached tissue around dental implants still remains controversial in the dental literature. A consensus report from the Consensus Conference of the European Association for Osseointegration ^39^ stated that “there is a lack of high‐quality studies evaluating the need for attached mucosa around implants to maintain health and tissue stability.” By contrast, recent systematic reviews demonstrated that a deficient band of attached tissue around implants is associated with greater plaque accumulation, mucosal inflammation (assessed by bleeding on probing), development of soft tissue recession, and patient discomfort while performing oral hygiene.^11^, ^13^, ^40^ This is further underlined by a recent systematic review indicating that soft tissue grafting procedures to gain attached mucosa resulted in a significantly greater improvement in gingival index values compared with maintenance groups (with or without attached tissue) and, for final marginal bone levels, statistically significant differences were calculated in favor of an apically positioned flap plus autogenous grafts vs all control treatments (apically positioned flap alone, apically positioned flap plus a collagen matrix, maintenance without intervention [with or without residual attached tissue]).^28^

### 3.2 Soft tissue volume

Soft tissue volume refers to the vertical and horizontal thickness of the peri‐implant tissues and is important for the formation of a biologic width around implants. Peri‐implant bone undergoes a remodeling process to allow sufficient space for the peri‐implant soft tissue to be formed.^41^, ^42^ The assessment of soft tissue volume is challenging because of the scarcity of measuring tools able to evaluate soft tissue changes. The introduction of digital optical scanning/analysis as an assessment method has allowed measurement of changes in soft tissue volume over time.^43^ Indications for mucosal volume augmentation include esthetic improvements, prevention of recession, facilitation of oral hygiene, and maintenance of marginal bone and peri‐implant health.

Horizontal tissue thickness (measured on the buccal side of the implant) has been associated with buccal tissue stability,^8^, ^44^ less marginal bone loss,^45^ and improved esthetic outcomes.^35^ Moreover, a sufficient vertical thickness of the mucosal tissues (measured coronal to the implant) has been associated with decreased marginal bone loss compared with thinner biotypes.^46^, ^47^ As shown in a recent systematic review,^28^ soft tissue grafting procedures for gain of mucosal thickness resulted in significantly less marginal bone loss over time.

### 3.3 Buccal soft tissue recession

Peri‐implant soft tissue recession can be a major esthetic complication, predominantly when occurring in the anterior maxilla. A number of factors appear to influence the level of the marginal mucosa.^36^ Those that have been shown to have a greater negative impact on the stability of the peri‐implant mucosa when they are not present are the quality of the mucosa (the presence of attached mucosa), the attachment levels of the adjacent teeth, and the thickness of the mucosa.^28^, ^48^

From an esthetic point of view, the gray color of the titanium implant and the implant components may create a major problem when they are exposed and visible as a result of peri‐implant mucosal recession.^49^, ^50^, ^51^ Unlike natural teeth, recession around implants with a minimal amount of titanium exposure can dramatically impact esthetic appearance,^52^ thus being unacceptable to the patient and requiring additional surgical and/or restorative treatment.

In addition, recession defects have also been associated with a deficient band of attached mucosa around the implant,^12^ and subsequently, a greater difficulty for patients to properly perform oral hygiene. When implant surfaces become exposed, especially for implants with a rougher surface,^53^ plaque accumulation will occur, thus potentially initiating the development of peri‐implant disease.

## 4 MANAGEMENT AND PREVENTION OF SOFT TISSUE COMPLICATIONS

The management and prevention of soft tissue complications are vital to prevent adverse outcomes in implant dentistry.

The selection of the type and time point of treatment depends upon the clinical characteristics of each case and the patient's wishes and needs. A thorough review of the patient's medical history, periodontal status, bone quality and quantity, and restorative needs should be performed prior to any soft tissue‐management procedure.

The proposed clinical decision tree is based on five time points during implant therapy:

(A) prior to implant placement (with or without tooth extraction).
(B) simultaneous with implant placement.
(C) within the implant‐healing phase (between implant placement and abutment connection).
(D) simultaneous with abutment connection.
(E) after delivery of the implant reconstruction.

This timing of treatment has not been thoroughly researched in the literature, but may exert a clinical impact on the final result of the implant reconstruction. Management of soft tissue conditions prior to delivery of the reconstruction (A, B, C, and D) can be considered as primary prevention of complications and may aid the clinician in achieving peri‐implant tissue stability. Once the restoration has been delivered (E), the treatment of these complications is more complicated and the predictability is reduced.^54^ Throughout this review, graded recommendations (highly recommended, recommended, or less recommended) will be given within text boxes for the time point of the treatment, depending on the indication and based on existing and, to some extent, limited evidence.

### 4.1 Attached and keratinized mucosa

Where there is a lack of attached mucosa, the preferred method of treatment is an apically positioned flap/vestibuloplasty procedure with or without the combination of a graft material.^19^ The use of autogenous transplants (free gingival graft or subepithelial connective tissue graft) is considered to be the gold standard, with a reported increase in attached mucosa ranging from 1.4 to 3.3 mm. Other therapeutic treatment modalities include the apically positioned flap/vestibuloplasty in conjunction with allogenic dermal matrix grafts or a collagen matrix. These options reduce treatment time and patient morbidity but are less well investigated.^19^ The therapeutic approach to increase the width of attached mucosa can be more predictably performed prior to implant placement (time point A). Augmentation of attached tissue dimensions to improve the quality of soft tissues does simplify the subsequent therapeutic steps, such as bone augmentation surgery or the insertion of a dental implant, thereby reducing the risk of tissue dehiscences with subsequent membrane/graft exposure. Based on a systematic review, it was recommended to address a lack of attached tissue at second stage surgery (time point D), where an apically positioned flap/vestibuloplasty in combination with a free gingival graft or a collagen matrix appeared to provide predictable outcomes.^20^

**Management of lack of attached mucosa:**

(A) before implant placement: highly recommended.
(B) with implant placement: less recommended.
(C) within the implant‐healing phase: less recommended.
(D) with abutment connection: recommended.
(E) after delivery of the implant reconstruction: less recommended.

### 4.2 Soft tissue volume

Soft tissue grafting procedures to increase mucosal thickness are successfully employed to eliminate soft tissue volume deficiencies around dental implants. For immediate implants, strong evidence suggests that implant placement and simultaneous hard tissue grafting should be combined with a soft tissue graft to counteract contour and remodeling processes following the surgical intervention. The addition of a subepithelial connective tissue graft demonstrated improved esthetics, as assessed by the pink esthetic score and less midfacial recession of the peri‐implant soft tissues.^55^, ^56^, ^57^

Dental implants may be placed early, delayed, or late. At these time points, remodeling processes may have already led to volume deficiencies. As such, following implant placement with or without concomitant guided bone regeneration, soft tissue volume grafting can be performed during second stage surgery. The combination of abutment connection and soft tissue grafting reduces the need for further surgical intervention. The previously mentioned systematic review^20^ determined that the use of an apically positioned flap in combination with a subepithelial connective tissue graft appeared to be a reliable treatment option to increase soft tissue volume during second stage surgery.

For delayed implants, a case series calculated that guided bone regeneration was responsible for 57%, and soft tissue grafting for 43%, of the total final volume.^58^ This indicates the importance of soft tissue grafting to enhance the final esthetic outcome. Autogenous tissue (subepithelial connective tissue graft) is considered the treatment of choice for soft volume augmentation around dental implants, resulting in an increase in soft tissue thickness in partially edentulous sites. Free gingival grafts have also been employed but with limited results and decreased color matching.^19^ More recently, soft tissue substitutes have been applied, serving as an alternative to a subepithelial connective tissue graft. Based on a randomized controlled clinical trial, employing either a subepithelial connective tissue graft or a newly developed collagen matrix, both treatment options resulted in an increase in soft tissue volume of up to 1.8 mm. However, the use of a newly developed collagen matrix reduced patient morbidity.^18^

**Management of lack of soft tissue volume:**

(A) before implant placement: recommended.
(B) with implant placement: highly recommended for immediate implants; less recommended for other time points.
(C) within the implant‐healing phase: highly recommended.
(D) with abutment connection: recommended.
(E) after delivery of the implant reconstruction: less recommended.

Later time points for volume or attached mucosa augmentation, especially after the insertion of the definitive reconstruction, are usually not included as part of the regular treatment, and are used instead to compensate for loss of quantity and/or quality of tissue occurring over time.^59^ These rescue treatments usually offer a decreased predictability and require more technique‐sensitive surgical skills.^54^

### 4.3 Buccal soft tissue recession

The midfacial mucosal level around a dental implant may be influenced by a wide range of clinical factors.^36^ Depending on the severity of the buccal mucosal dehiscence, treatment approaches include mucogingival surgery, the replacement of the crown, or even removal of the implant. Once the implant restoration is in place and recession occurs, management poses a greater challenge for the clinician. Even although there is no direct cause‐and‐effect evidence, there is a clinical understanding that the amount of coverage of the soft tissue augmentation surgery will be governed by interproximal attachment of the adjacent teeth, the presence of bone on the buccal surface of the implant, the horizontal and vertical implant positions, and the thickness of the peri‐implant mucosa.

Only a few prospective studies have evaluated mucogingival surgical procedures to correct mucosal recessions.^16^, ^54^, ^59^ The results, based on the use of coronally advanced flaps in combination with a subepithelial connective tissue graft in all three studies, showed coverage of the recession ranging from 66% to 96%. The implants studied were treated when they were single healthy implants. In a systematic review,^60^ it was concluded that mucosal recessions can be treated with an expected gain of 1.6 mm in vertical soft tissue height, but without any long‐term evidence of the stability of the tissues. However, midfacial peri‐implant soft tissue recession coverage is less successful than recession coverage around natural teeth, with Miller recession class I and II being very predictably treated and maintained.^61^

Certain factors should be evaluated before treatment, such as the presence of buccal bone, the attachment of the adjacent teeth, the implant position, the emergence profile of the reconstruction, and the tissue biotype. Treatment is indicated for single healthy implants within their bony housing, where the implant position is correct and where adjacent teeth have a well‐maintained periodontium. There are cases when such surgery is indicated. Overcontoured restorations with emergence profiles impinging on the soft tissue should be changed to provide space for the tissue to develop. When the implant is placed in an exaggerated buccal position outside the bony housing and a buccal dehiscence occurs, the only possible treatment is removal of the implant.

**Management of soft tissue recession:**

(A) before implant placement: not possible.
(B) with implant placement: not possible.
(C) within the implant‐healing phase: not possible.
(D) with abutment connection: recommended.
(E) after delivery of the implant reconstruction: recommended.

## 5 CLINICAL CONCEPTS FOR MANAGEMENT AND PREVENTION OF SOFT TISSUE COMPLICATIONS AROUND IMPLANTS

**FIGURE 1:**
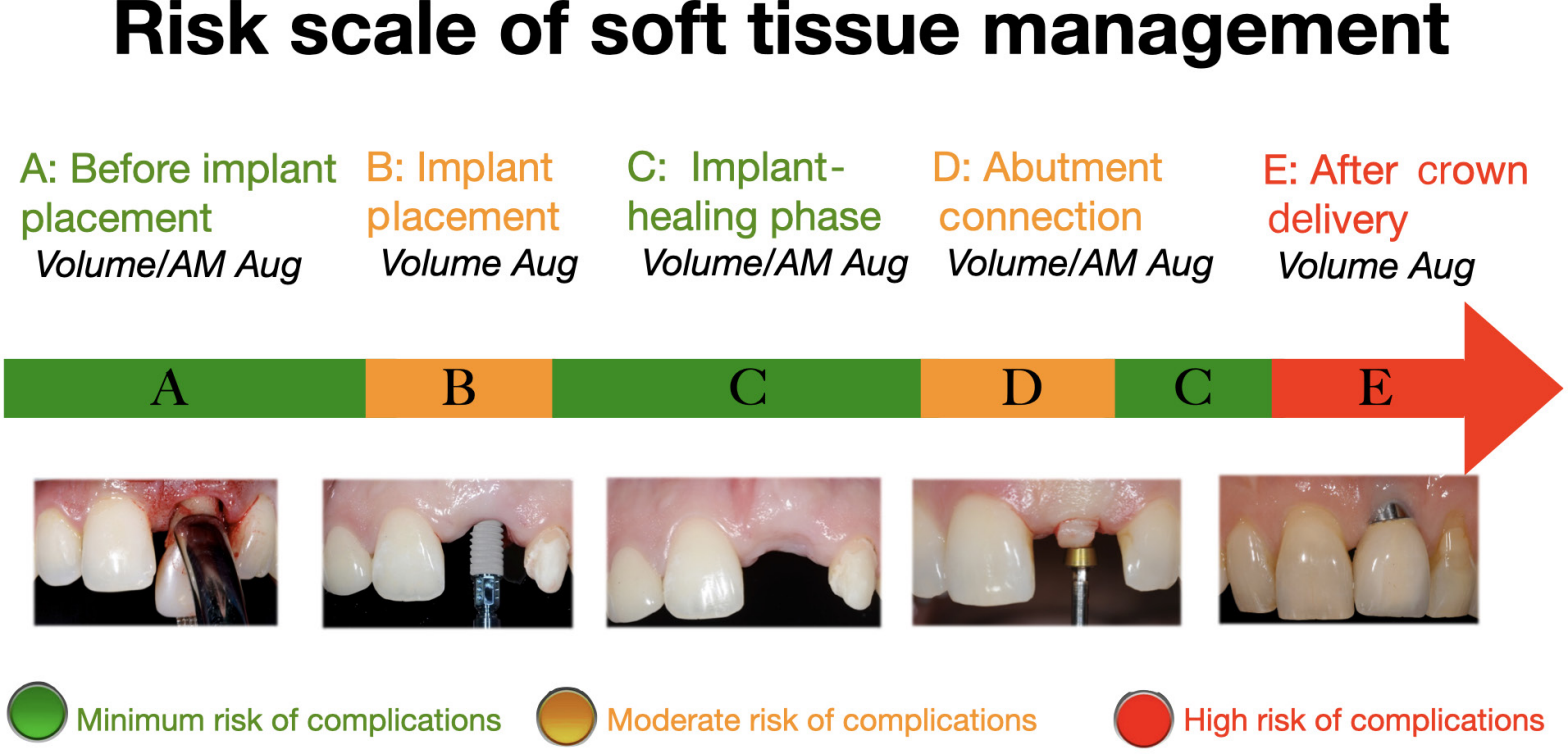
Risk scale of soft tissue management before, during, and after implant placement. AM, attached mucosa; Aug, augmentation

The clinical concepts as presented in the current review is based on a risk assessment of the peri‐implant tissues encompassing different time points for the prevention and management of soft tissue complications around implants. Depending on the stage of treatment, different approaches can be performed, with differing levels of success and predictability (Figure 1).

Bearing in mind that the concept presented lacks scientific evidence, the predictability of surgical and prosthetic treatment appears to be higher if performed during earlier stages of implant therapy. That said, managing complications after delivery of the definitive reconstructions is considered to be the least predictable. It is also suggested that the number of procedures (eg, hard and soft tissue grafting) per time point is limited to increase the predictability of soft tissue management.

### 5.1 Clinical concept for prevention of soft tissue complications before implant placement

Treatment planning encompassing dental implants should include a risk assessment of the soft tissue situation prior to implant surgery. If there is any type of soft tissue deficiency (such as a lack of attached mucosa and/or lack of volume), this should be addressed before any surgery is undertaken at the level of the bone. Once the condition of the edentulous mucosa is ideal, implant‐related surgery can be performed with increasing predictability.

**FIGURE 2:**
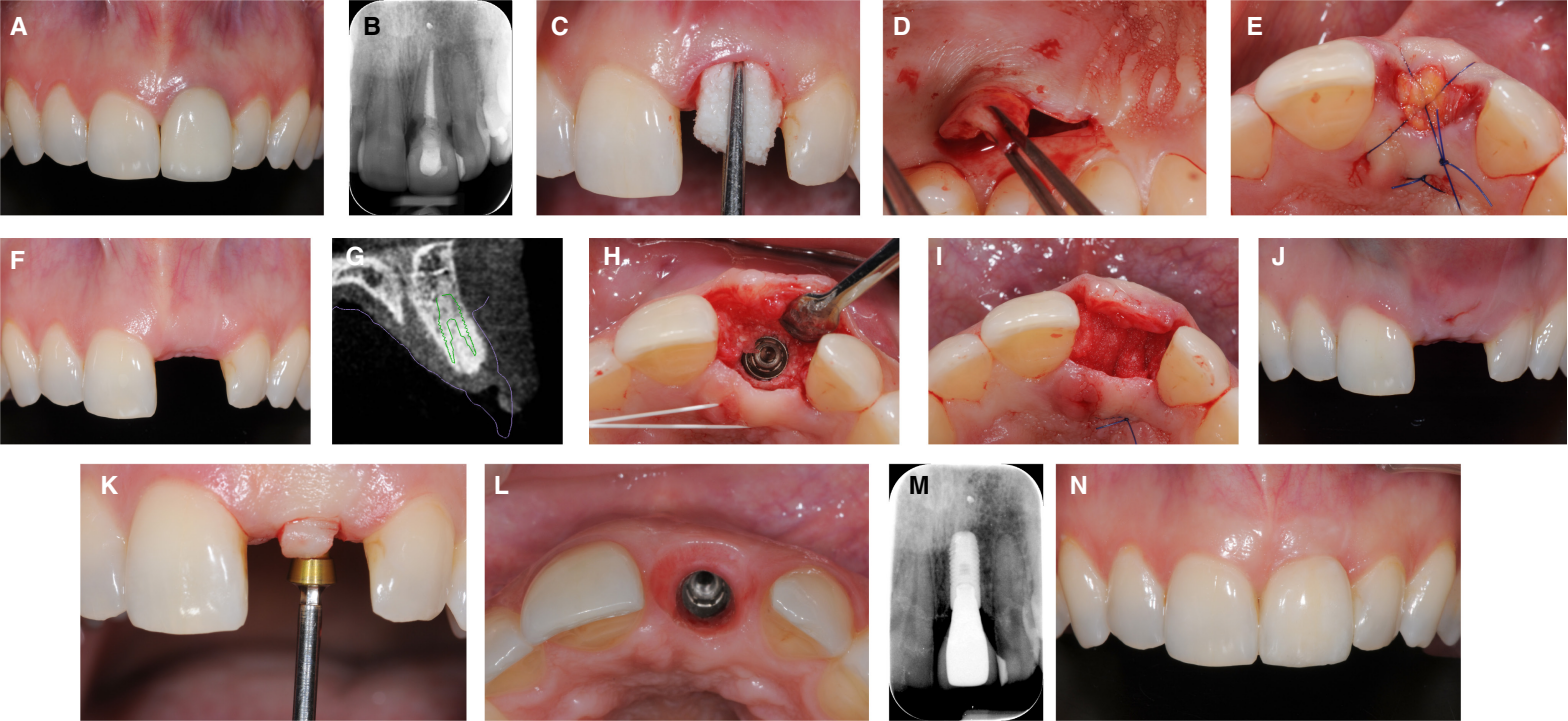
Clinical case of soft tissue management simultaneous to ridge preservation and in combination with implant placement. A, Patient's initial situation. B, Periapical radiograph of tooth 21. C, Alveolar ridge preservation. D, Palatal sub‐epithelial connective tissue graft. E, Sealing of the socket in ridge preservation. F, Post‐operative healing after 6 months. G, Cone‐beam computed tomography scan of healed site 21. H, Implant placement. I, Soft tissue grafting with volume‐stable xenogeneic collagen matrix. J, Post‐operative healing after 2 weeks. K, Abutment connection with U‐Flap. L, Emergence profile of implant 21. M, Periapical radiograph of osseointegrated implant 21. N, Patient's final situation after receiving the implant supported‐restoration

A 32‐year‐old patient (Figure 2) presented with root resorption at a central incisor (tooth 21) (A,B) following trauma 10 years previously. The tooth was extracted and ridge preservation was performed with the use of a xenograft (C) and autogenous connective tissue from the palate (D,E). The grafted area healed uneventfully (F), and the edentulous area showed sufficient tissue height, no invagination, but a slight volume deficiency on the buccal aspect at 6 months. A cone beam computed tomography scan showed sufficient bone to place a dental implant, without the need for further bone augmentation (G). An implant was placed (H) and a volume‐stable xenogeneic collagen matrix (Fibro‐Gide, Geistlich Pharma AG, Wolhusen, Switzerland) was placed on the crestal and buccal side of the implant to increase the tissue thickness and to compensate for the missing tissue volume (I). The situation after 2 weeks of healing demonstrated increased height and bucco‐oral width of the soft tissue (J). A U‐flap was performed during abutment connection to mobilize the tissue to the buccal side (K). An adequate emergence profile with increased horizontal thickness was created through the use of a provisional restoration (L). The definitive reconstruction was a screw‐retained ceramic reconstruction. The situation postdelivery shows stable bone levels (M) and stable soft tissue conditions (N).

**FIGURE 3:**
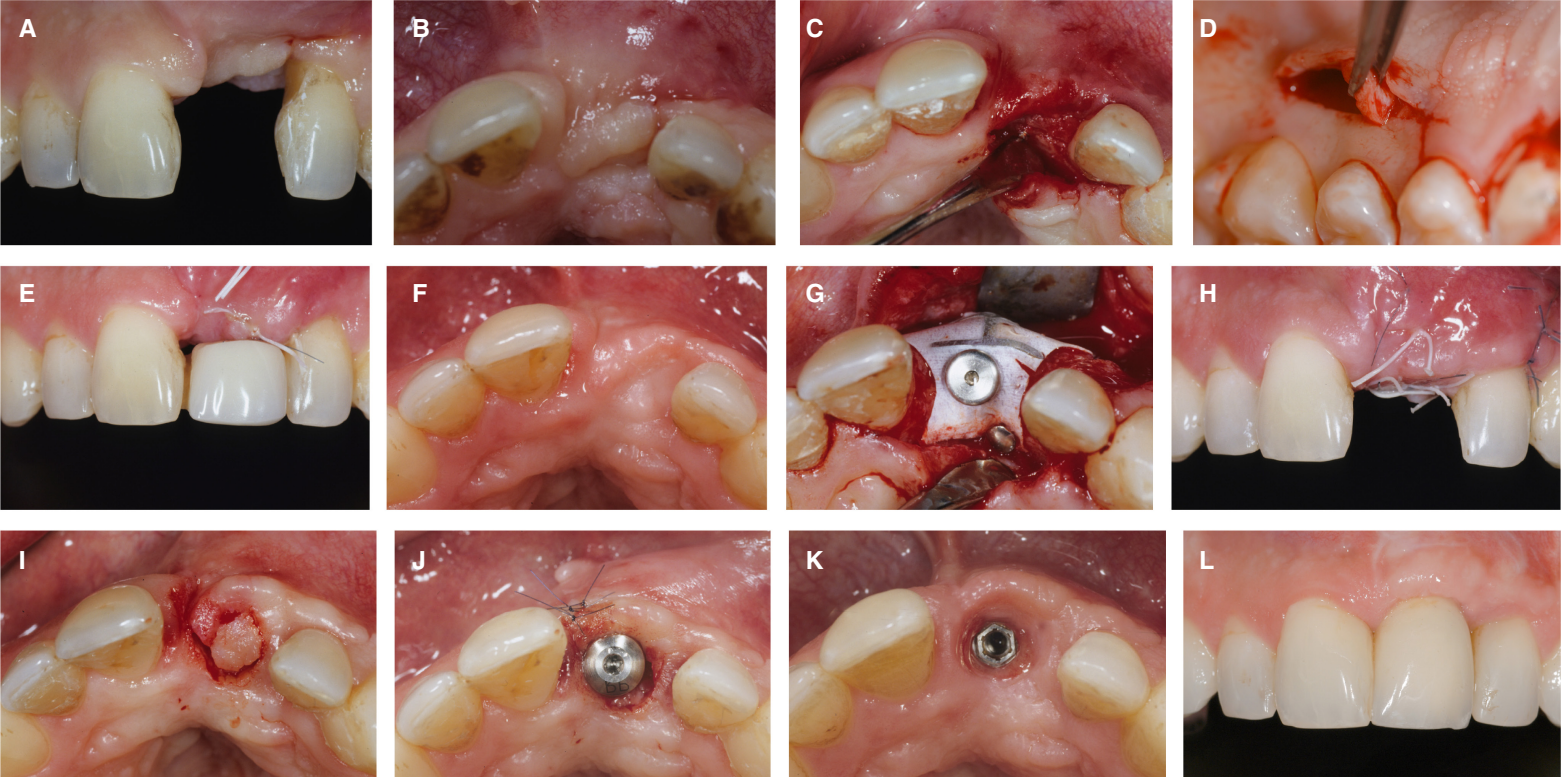
Clinical case of soft tissue management prior to implant and guided bone regeneration surgery. A‐B, Patient's initial situation. C, Minimally‐invasive flap combined with tunneling technique. D,Palatal sub‐epithelial connective tissue graft. E, Suturing with resin bonded provisional. F, Post‐operative healing after 8 weeks. G, Guided bone regeneration simultaneous to implant placement. H, Flap closure with sutures. I, Abutment connection with minimal U‐Flap. J, Abutment connection with healing abutment. K, Emergence profile of the implant. L, Seven year follow‐up

A 36‐year‐old patient (Figure 3) presented with a missing tooth 21 with deficient soft tissues in the edentulous area 21 and at the adjacent tooth 22 (A,B). There was a lack of volume, a lack of attached and keratinized tissue, deep soft tissue invaginations, and severe attachment loss at the neighboring tooth 22. Prior to any surgical intervention at the level of the bone, a preimplant soft tissue grafting procedure was considered mandatory. Therefore, the edentulous area was grafted with subepithelial connective tissue from the palate (D) using a combination of a limited flap with a tunneling technique (C). Following graft stabilization, the tissue was coronally positioned with sutures (E) and a resin‐bonded provisional crown was delivered. Healing after 8 weeks demonstrated an increase in volume with an improved soft tissue situation (F). An implant was then placed with a simultaneous guided bone regeneration approach (G) with the use of a xenograft and a nonresorbable membrane. The sutures were removed 10 days later. At this time point, the tissues had healed in a more coronal position (H). The implant was left to heal submerged for 6 months. After healing, abutment connection was performed with a U‐flap (I) that further increased the soft tissue volume on the buccal side (J). An adequate emergence profile with sufficient soft mucosal thickness and attached mucosa and even partial root coverage (K) was obtained once the definitive crown was delivered 4 months later. The situation remained biologically stable and esthetically pleasing at the 7‐year follow‐up (L).

**FIGURE 4:**
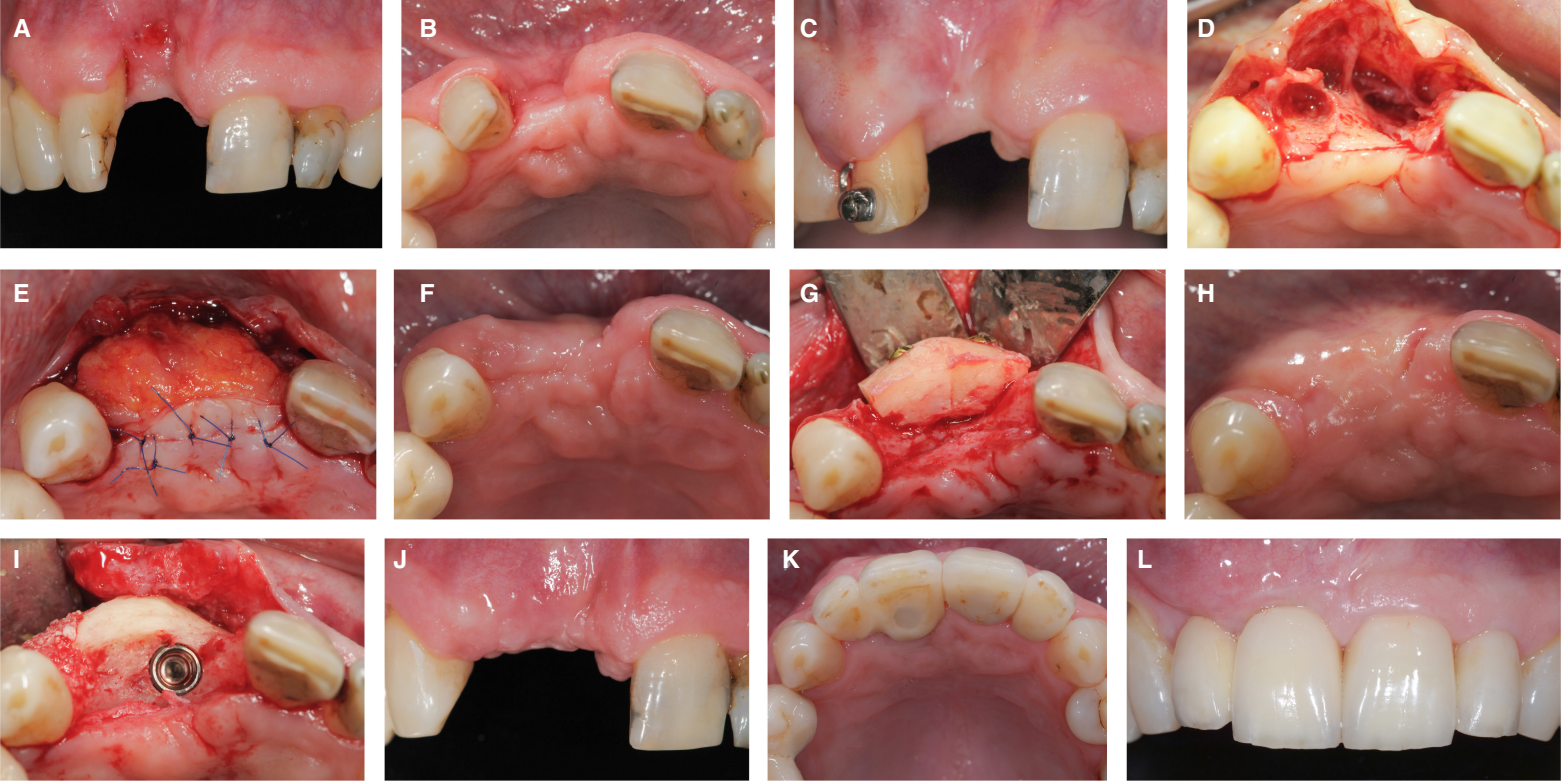
Clinical case of soft tissue management prior to tooth extraction and prior to block bone graft and posterior implant placement. A‐B, Patient's initial situation. C, Orthodontic extrusion on tooth 12 after 6 months. D, Extraction of tooth 12. E, Palatal subepithelial connective tissue graft stabilization. F, Post‐operative healing after 6 weeks. G, Bone augmentation with an autogenous block bone graft. H, Post‐operative healing after 4 months. I, Implant placement 11. J, Post‐operative healing after 3 months. K, Implant‐supported restoration 11 with a distal cantilever 12. L, Clinical situation after y ears of delivery of the restoration

A 46‐year‐old patient presented with a missing tooth 11 (Figure 4) and severe attachment loss at the mesial aspect of the neighboring tooth 12 (A). The soft tissue deficit was severe, in both the horizontal and vertical dimensions in the edentulous area. Moreover, deep interproximal recession as a result of the loss of attachment was present at the adjacent lateral incisor 12 (B). The priority in this case was to improve the soft tissue condition until a stable situation could be achieved. Accordingly, orthodontic extrusion was performed on tooth 12 to regain the missing mesial attachment. After 6 months of extrusion (C), the interproximal tissue had advanced coronally and there was substantial clinical attachment level gain. Tooth 12 was then extracted (D) and a subepithelial connective tissue graft was harvested from the palate and stabilized in the recipient site (E). The healing after 6 weeks demonstrated a significantly increased mucosal thickness (F). Subsequently, bone augmentation was performed by means of an autogenous block graft harvested from the mandibular symphysis. The block was stabilized with two screws (G) and left to heal for 4 months. The healing was uneventful and the 4‐month follow‐up situation showed a ridge with increased volume (hard and soft tissue) (H). A dental implant was placed in position 11 with simultaneous guided bone regeneration using a xenograft and a resorbable collagen membrane (I). After 3 months of submerged healing (J), abutment connection was performed and the emergence profile was created by use of a provisional reconstruction. The implant reconstruction (position 11) was screw‐retained with a distal cantilever for site 12 (K). Clinical pictures 4 years after the delivery of the reconstruction demonstrated healthy and stable tissue and a pleasing esthetic outcome (L).

### 5.2 Clinical concept for prevention of soft tissue complications with implant placement

There are situations when the soft tissue condition is inadequate in volume following tooth extraction but without being sufficiently deficient to contraindicate implant placement. In this situation, the soft tissue augmentation surgery can be performed at the same time as implant placement, provided that the graft can be stabilized over the implant and no additional guided bone regeneration is needed. If the implant site is lacking attached tissue, the soft tissue augmentation surgery should be performed prior to implant placement or during the implant‐healing period, but not simultaneously because of the difficulty in stabilizing the graft.

**FIGURE 5:**
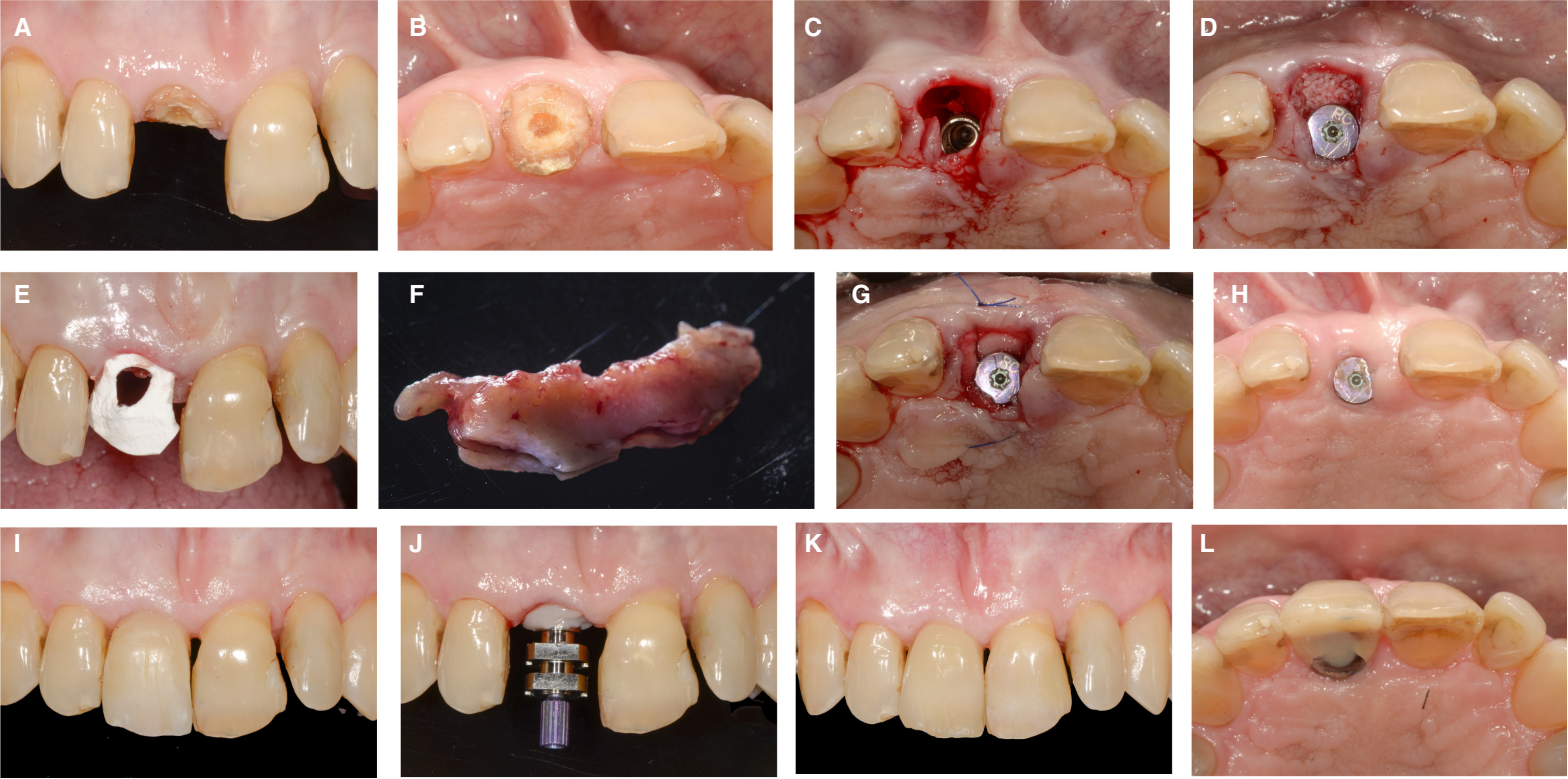
Clinical case of soft tissue management simultaneous to implant placement and guided bone regeneration. A‐B, Patient's initial situation. C, Immediate implant placement after extraction of tooth 11. D, Guided bone regeneration performed buccal to implant 11. E, Collagen membrane adapted to seal the socket. F, Sub‐epithelial connective tissue graft. G, Partial thickness pouch buccal to the implant with autogenous graft. H, Post‐operative healing after 3 months. I, Implant‐supported provisional restoration. J, Conventional open tray impression of implant 11. K‐L, Implant‐supported restoration on 11 after one year follow‐up

A 65‐year‐old patient presented (Figure 5) with a fractured central incisor (tooth 11) (A,B). After analyzing the bone dimensions (sufficient palatal bone) and the favorable soft tissue condition (adequate interproximal attachment levels), an immediate implant was planned simultaneously with the extraction. The tooth was extracted with caution to preserve the buccal plate. A bone‐level implant was placed into the palatal cortical bone, in a screw‐retained position (C). The gap between the implant and the buccal bone was grafted with a particulate xenograft (D) and a resorbable collagen membrane was adapted and used to cover the exposed bone graft (E). An autogenous connective tissue graft was harvested from the tuberosity (F) and was placed in a previously created partial thickness pouch, buccal to the implant (G). A healing abutment was placed and the implant was left to heal for a period of 4 months. The healing after 3 months demonstrated a favorable tissue thickness on the buccal side (H). The patient was provided with a provisional restoration to wear for another 3 months to shape the emergence profile of the implant (I). Once the desired shape of the mucosa was achieved, an open tray impression was performed (J) to fabricate the definitive reconstruction. The final situation 1‐year postdelivery of the porcelain fused to metal reconstruction is illustrated from both a buccal (K) and occlusal perspective (L).

### 5.3 Clinical concept for prevention of soft tissue complications within the implant‐healing phase

During the implant‐healing period, the soft tissue condition can be easily assessed for any lack of volume or lack of attached tissue. Performing the soft tissue augmentation on its own allows for an undisturbed healing phase following implant placement, with or without bone augmentation. Therefore, the use of partial thickness flaps allows for stabilization of the soft tissue grafts without affecting the implant or the bone healing.

**FIGURE 6:**
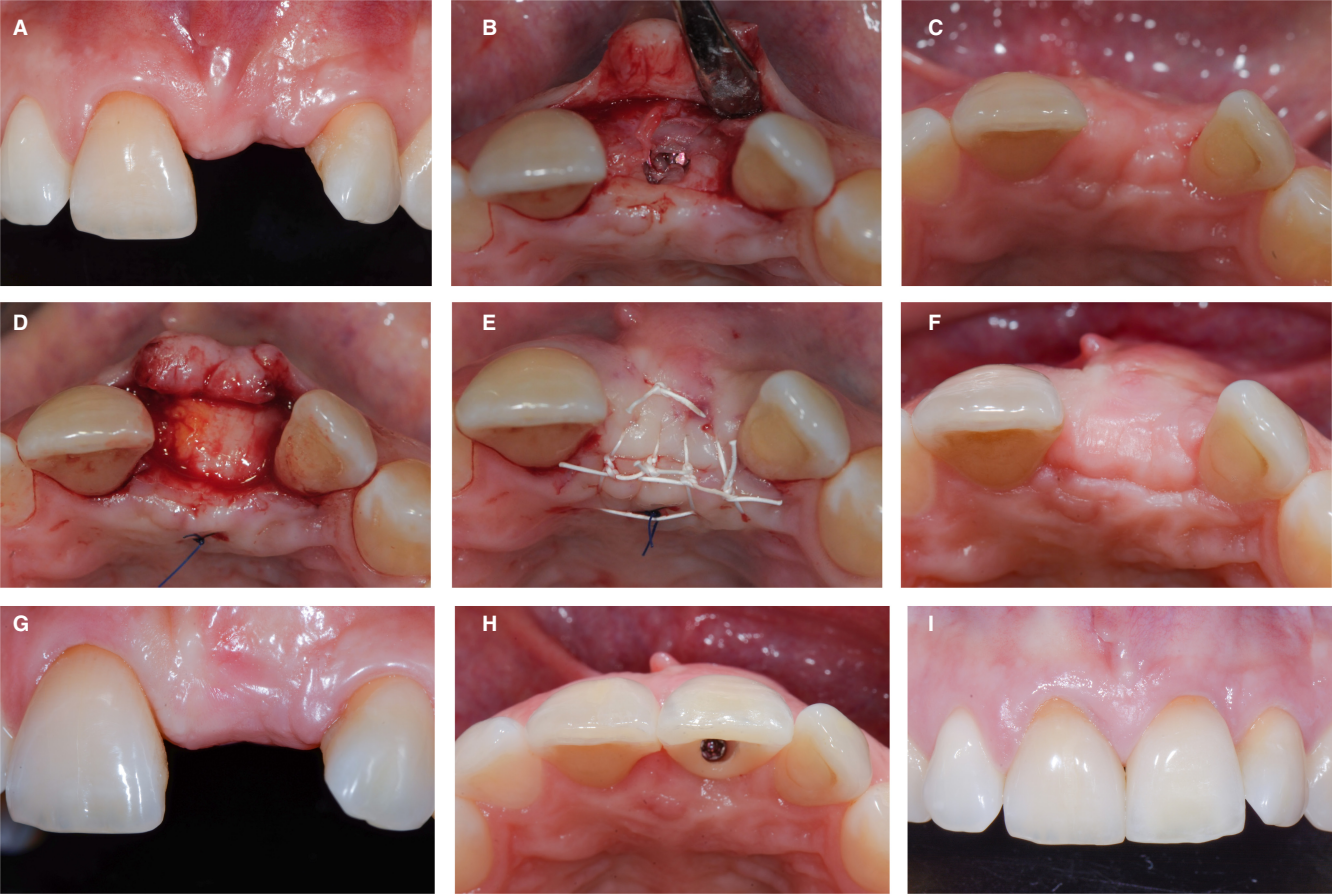
Clinical case of soft tissue management within the implant healing phase, after implant placement and before abutment connection. A, Patient's initial situation with missing tooth 21. B, Implant placement without augmentation on 21. C, Post‐operative healing after one month. D, Placement of a sub‐epithelial connective tissue graft buccal and occlusal to the implant. E, Primary wound closure with sutures. F‐G, Post‐operative healing after 2 months. H, Implant‐supported restoration on 21. I, Clinical situation at three year follow‐up

A 22‐year‐old patient presented with a missing tooth 21 (Figure 6) as a result of a failed root canal treatment (A). An implant was placed without any augmentation procedure (B). The healing after 1 month showed a deficiency in buccal tissue volume (C). A soft tissue augmentation was performed using a connective tissue graft harvested from the palate and by the use of a partial thickness flap (D). The graft was stabilized with sutures on the palatal side and positioned coronally and buccally. The flap was then sutured with a tension‐free mattress suture and single interrupted sutures (E). Healing after 2 months was uneventful and showed increased thickness in both the horizontal (F) and vertical dimensions (G). At the point of delivery of the reconstruction, sufficient buccal tissue volume had been created and formed an emergence profile mimicking the one of the contralateral tooth site (H). The follow‐up situation after 3 years showed a stable peri‐implant mucosa with a natural and esthetically pleasing result (I).

### 5.4 Clinical concept for prevention of soft tissue complications during abutment connection

Soft tissue grafting procedures are commonly performed in conjunction with abutment connection. Because of the need for a surgical intervention (abutment connection), surgical procedures to gain attached mucosa as well as to provide mucosal thickness can be addressed at the same time point. Whereas in the esthetic zone a lack of volume is often observed, in the nonesthetic area surgical procedures usually address a lack of attached mucosa.

**FIGURE 7:**
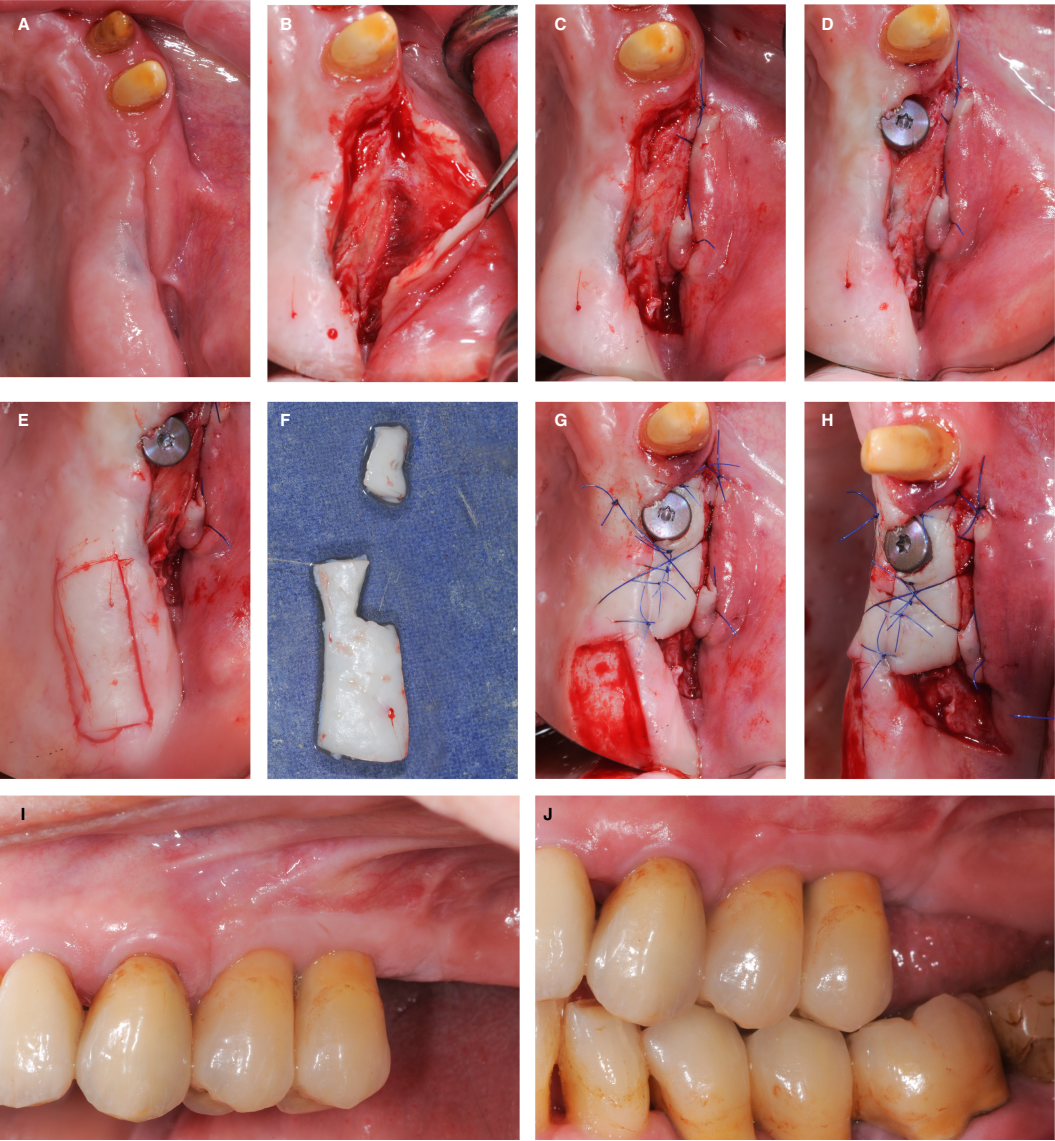
Clinical case of soft tissue management at abutment connection. A, Patient's initial situation. B, Partial thickness incision buccal to placed implant 24. C, Apical suturing of the flap. D, Healing abutment placed on implant 24. E, Free gingival graft from the palate. F, Trimming of the free gingival graft. G‐H, Stabilization of the free gingival graft with sutures. I, Post‐operative healing after 4 weeks. J, Clinical situation after 2 years of follow‐up

A 55‐year‐old patient presented with partial edentulism (Figure 7). She had undergone multiple extractions; one implant had been placed (site 24) and subjected to submerged healing in the maxillary left posterior quadrant (A). During abutment connection, a deficient soft tissue condition (with a shift of the mucogingival junction towards the palate) was observed. A partial thickness incision was raised distal to the abutment tooth (B). The flap was then sutured apically and secured with periosteal sutures (C). The implant cover screw was removed and the healing abutment placed (D). A free gingival graft was harvested from the palate (E). The graft was trimmed to fit the recipient site (F) and was then secured with sutures to the palatal side of the flap (G). The additional use of overlapping cross sutures stabilized the graft on the wound bed (H). After 4 weeks of healing, the recipient site was well integrated and showed a significant increase in the width of attached mucosa (I). After 2 years, the grafted site still demonstrated sufficient width of attached tissue with no signs of scar formation (J).

### 5.5 Clinical concept for management of soft tissue complications following delivery of the reconstruction

Once the implant reconstruction is delivered, any soft tissue deficiencies (considered a complication) will be less predictable to treat. The management of such complications is still possible in some cases. Usually, the most noticeable type of soft tissue complication following implant crown delivery is the development of a recession on the buccal side of the implant. This can have a very negative impact on the esthetic appearance of the reconstruction and may be considered a failure by the patient. The treatment of such buccal mucosal dehiscences depends on multiple factors including the implant position and depth, the tissue phenotype, and the periodontal attachment of the adjacent teeth. When the implant is correctly positioned, and the periodontal attachment of the adjacent teeth is preserved, soft tissue augmentation surgery can be performed to improve the situation. Other treatment options include changing the reconstruction, or even removal of the implant.

**FIGURE 8:**
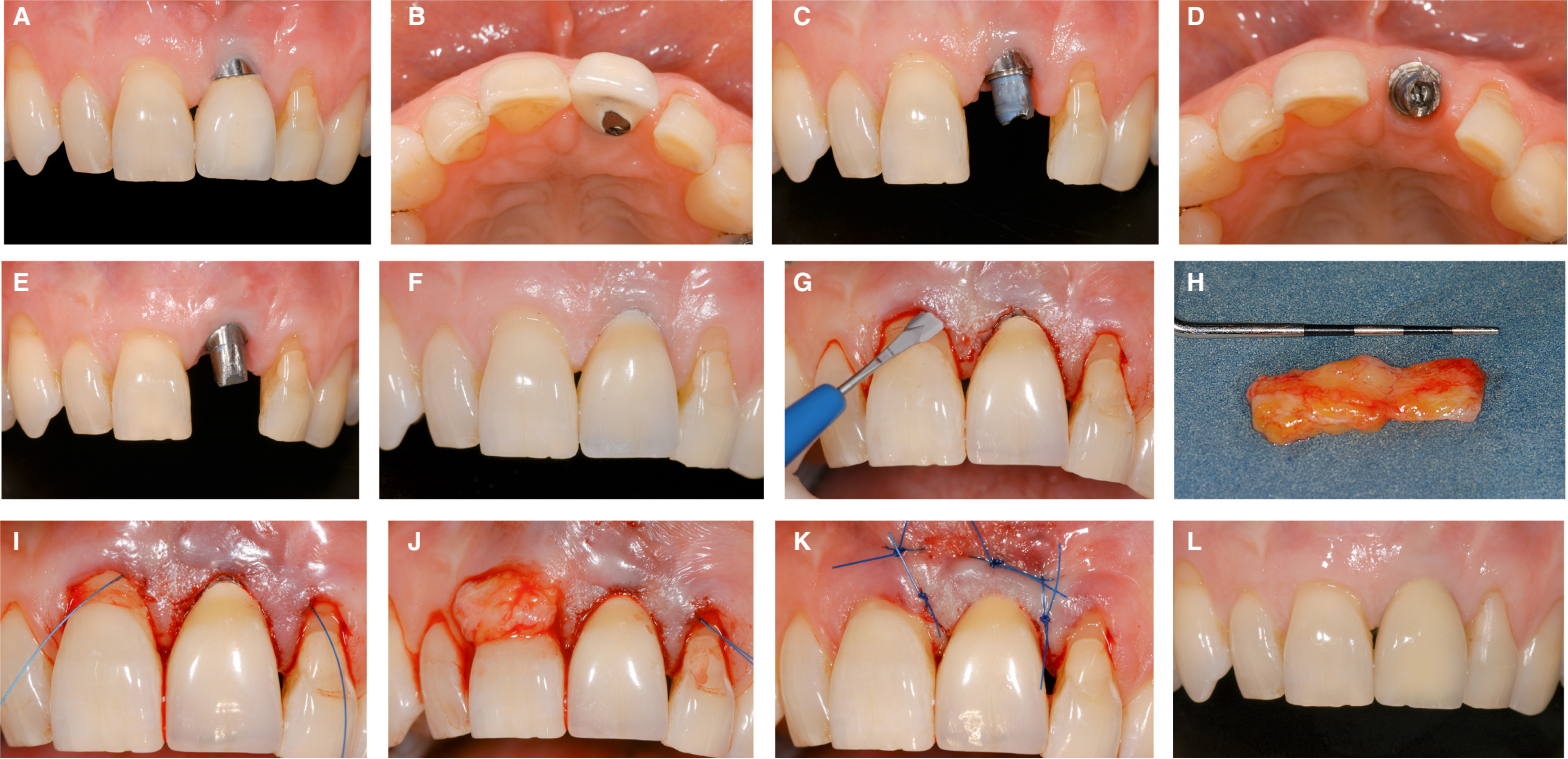
Clinical case of soft tissue management after the delivery of the implant reconstruction. A, Patient's initial situation with implant recession on 21. B, Patient's initial situation with reduced buccal volume. C‐D, Implant restoration removed. E, Implant abutment polished. F, Modification of implant‐supported restoration 21. G, Tunneling procedure around implant mucosa and adjacent teeth. H, Palatal sub‐epithelial connective tissue graft. I, Placement of anchorage sutures. J, Stabilization of graft inside the tunnel with anchorage sutures. K, Coronal advancement of the tunnel with sutures. L, Clinical situation after 2 years follow‐up

A 46‐year‐old patient presented (Figure 8) with an esthetic concern attributable to the gray appearance of the implant caused by a buccal dehiscence of the peri‐implant soft tissues (A). This dehiscence had developed following delivery of the final implant‐borne reconstruction. An occlusal view of the situation shows a lack of soft tissue volume buccal to the implant (B). Initially, the crown was removed (C,D) and the implant abutment was reduced in thickness and polished to allow for better soft tissue adaptation (E). The reconstruction was also modified to cover the metal part of the abutment and to improve the situation (F). Nevertheless, the lack of tissue thickness in the buccal area had to be addressed through soft tissue augmentation surgery. A tunnel was created through the sulcus of the adjacent teeth (G). A thick connective tissue graft from the palate was harvested (H). Anchorage sutures were placed through the sulcus (I) and the graft was then introduced through the tunnel and stabilized with sutures (J). The situation after placement of the sutures (K) shows a coronal advancement of the tunnel with the graft in place on the buccal side. After 2 years, the implant recession coverage remained stable with increased thickness of the peri‐implant tissues (L).

**FIGURE 9:**
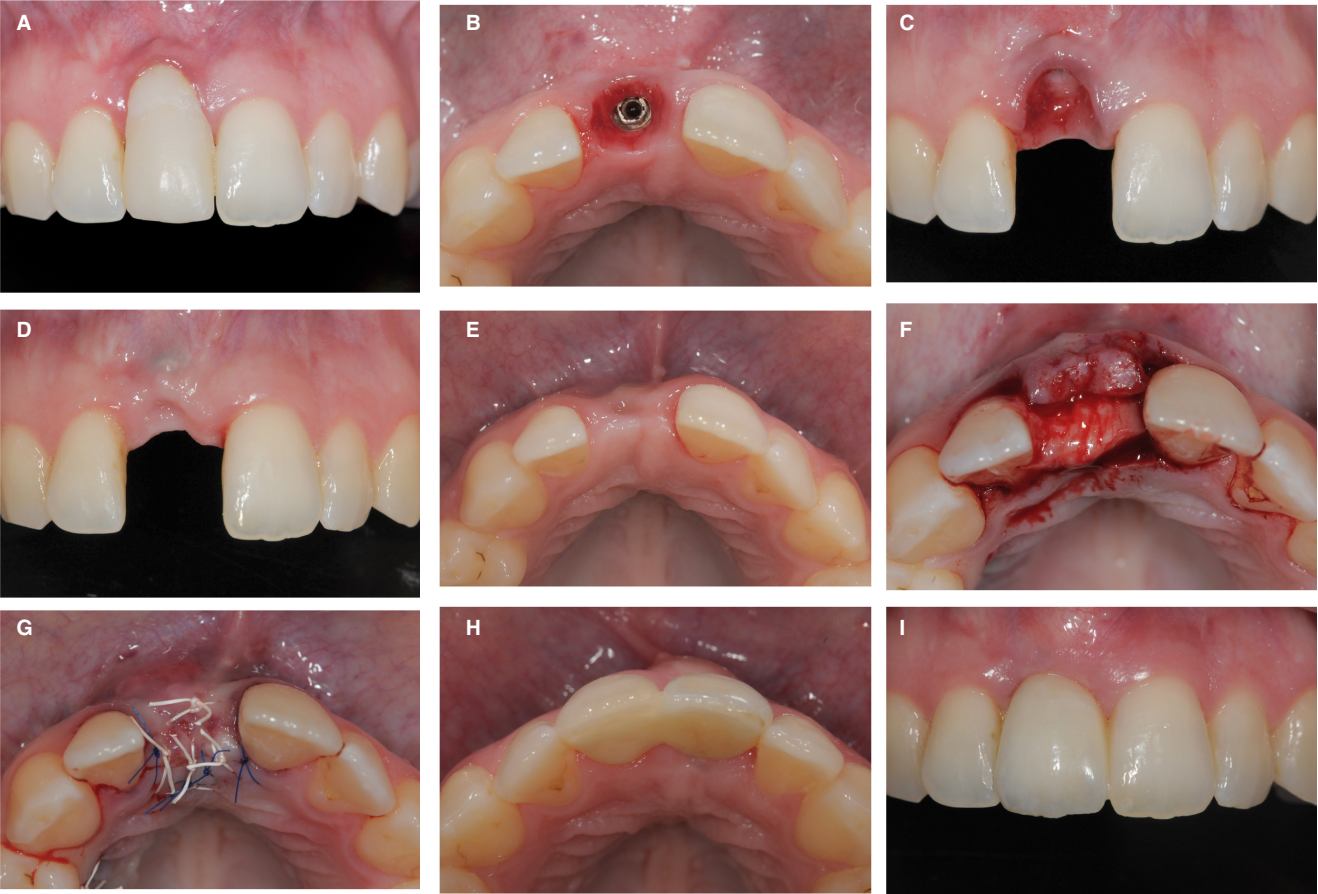
Clinical case of soft tissue management after the delivery of the implant reconstruction. A, Patient's initial situation with soft tissue margin discrepancy. B‐C, Removal of the implant‐supported restoration 11. D, Healing after 4 weeks without the restoration 11. E, Soft tissue volume deficiency on site 11. F, Stabilization of a sub‐epithelial connective tissue graft buccal and occlusal to the implant. G, Primary wound closure with sutures. H, A resin bonded bridge cemented on tooth 11. I, Clinical situation after 3 year follow‐up

A 38‐year‐old patient was (Figure 9) very dissatisfied with the esthetic result of an implant‐supported restoration on 11 position (A). After removing the reconstruction (B), various soft tissue predictive factors were analyzed, such as the position of the implant, the thickness of the mucosa, and the level of attachment of the neighboring teeth (C). Because of the excessive buccal position of the implant, it was decided that soft tissue augmentation surgery was not indicated and the tissue was left to heal without the implant‐retained crown in place. Following an initial healing period, the mucosa was positioned at a more coronal level (D). Nevertheless, there was still a significant lack of volume from the horizontal aspect in site 11 (E). A connective tissue graft was harvested and secured with sutures on the crestal and buccal side of the implant and left to heal submerged (F,G). A resin‐bonded bridge was then fabricated and utilized as the means of reconstruction (H). The esthetic result was significantly improved with no graft surgery. The outcome remains stable after 3 years (I).

## 6 DISCUSSION

The prevention and management of soft tissue complications is of key importance in modern implant dentistry. Therefore, assessment of soft tissue conditions from a quantitative and qualitative perspective should be part of the overall treatment plan. Such an assessment will dictate whether soft tissue management is needed and, if indicated, when the ideal time point would be to perform such a procedure. This approach helps clinicians to prevent soft tissue complications that can jeopardize both biologic and esthetic outcomes.

Cases with peri‐implant soft tissue complications generally involve: (a) a lack of attached and keratinized tissue; (b) insufficient volume; (c) development of mucosal dehiscences; or (d) a combination of (a), (b), and (c):

- A lack of attached mucosa has been associated with greater plaque accumulation, greater mucosal inflammation, higher chances of developing soft tissue recession defects, and difficulties in performing proper maintenance for the patient.^11^, ^13^, ^17^, ^40^
- A lack of soft tissue volume can have a crucial impact on the final results of the implant reconstruction. Mucosal thickness has a significant influence on color changes of the mucosa^35^, ^62^ and plays a crucial role in soft tissue esthetics.^34^ Moreover, thin soft tissues have been demonstrated to have a negative effect on marginal bone levels^28^, ^47^ and present a greater risk of developing recession.^44^
- The development of a peri‐implant buccal soft tissue recession defect can expose the gray color of the abutment or of the implant and cause an esthetic complication to the reconstruction.^16^, ^52^, ^54^ Unlike teeth, where minimal recession does not always result in an esthetic concern, patients do not accept the persistence of even minimal recession at the implant site following therapy.^59^ In addition, the exposure of the implant creates a favorable environment for plaque accumulation and biofilm formation, which may lead to potential development of peri‐implantitis.

Recent evidence has shown the importance of a healthy peri‐implant mucosa, both from a biologic^19^ and an esthetic perspective.^16^ According to a current systematic review,^28^ soft tissue grafting procedures can be recommended for gain of attached tissue and increase of mucosal thickness. For the former, the use of an apically positioned flap in conjunction with autogenous grafts results in greater improvement compared with sites without a surgical intervention. For the latter, the use of autogenous grafts is generally recommended and results in more stable marginal bone levels.

Apart from the importance of the different grafting techniques, the timing of these particular interventions is important and has an impact on the overall risk of the therapy. Consideration should be given to the quantity and quality of both the attached mucosa and the mucosal thickness present. It is generally understood in cases of a lack of attached tissue or mucosal volume that an earlier surgical intervention results in greater predictability.

Concept‐wise, the ideal time point to manage deficient soft tissue defects is considered to be prior to implant placement. Several authors have described techniques to address the soft tissues at the time of tooth extraction^63^, ^64^, ^65^ and various techniques have been described; among these are the use of a free gingival graft or a connective tissue graft harvested from the palate to optimize the soft tissues in the short term. This time point of management allows for an improvement in soft tissue condition (the presence of a wide band of attached tissue and a thick mucosa horizontally and vertically) before any surgery is performed at the level of the bone. High predictability and reliability for a pleasing esthetic result can be expected for future type 2 or type 3 implant placement.

The second most optimal time point for soft tissue management is during the healing phase after implant placement. Following implant installation, clinicians usually wait for 2‐4 months before loading the implant. If it is perceived that the peri‐implant mucosa is deficient, soft tissue grafting procedures can be performed as a single intervention, thereby not hampering the healing of the augmented bone beneath or the soft tissue graft itself. Keeping the implant submerged during healing enables proper positioning and stabilization with sutures of the graft buccal and/or crestal to the implant.

The third most optimal time point is simultaneous with implant placement or at the time of abutment connection. Recent studies have shown that with immediate implant placement it is beneficial to use a connective tissue graft to thicken the buccal contour. This allows for a change of the phenotype at the implant site and limits the development of recession and esthetic deficiencies.^56^, ^57^, ^66^ A systematic review analyzed soft tissue augmentation procedures performed during abutment connection surgery.^20^ It was concluded that, for the increase of peri‐implant attached and keratinized tissue, an apically positioned flap/vestibuloplasty in the maxilla, and an apically positioned flap/vestibuloplasty in combination with a free gingival graft or xenogeneic graft material in the mandible, both appeared to provide favorable outcomes. To increase the soft tissue volume, a roll envelope flap in the maxilla or an apically positioned flap plus a connective tissue graft in the mandible appear to be the most predictable treatment options.

The simultaneous approach (immediate implant or combining abutment connection in conjunction with soft tissue grafting) reduces the morbidity of the treatment by sparing the patient additional surgery. Limitations apply if the implant or the abutment could interfere with the stabilization and proper positioning of the soft tissue graft. The predictability of the soft tissue augmentation procedure may therefore decrease.

The least ideal time for soft tissue augmentation is following insertion of the final reconstruction. This time point is not considered as part of the treatment plan and is usually performed to compensate for severe tissue deficiencies. It can be regarded as a “rescue treatment,” is associated with decreased predictability, and is highly technique‐sensitive.^54^, ^59^ Quite often, the restoration needs to be removed to perform the augmentation surgery. This creates an additional need to provide the patient with a temporary solution, thereby increasing the treatment costs and time.

## 7 CONCLUSION

The quality and quantity of peri‐implant soft tissues are crucial factors and significantly influence biologic and esthetic outcomes in implant dentistry. A proper risk assessment and management of the soft tissues at the planned implant site is important prior to any implant‐related surgery. In cases of soft tissue deficiencies, these should be addressed as early as possible to increase the predictability of the surgical interventions.
